# Spontaneous renal fornix rupture in pregnancy and the post partum period: a systematic review of outcomes and management

**DOI:** 10.1186/s12894-020-00660-z

**Published:** 2020-08-04

**Authors:** Matthew McKnoulty, Ayla Green, Susan Scott, Matthew J. Roberts, Alka Kothari

**Affiliations:** 1 Faculty of Medicine, University of Queensland, Herston, 4006 Australia; 2 Queensland University of Technology, AUSHI, Kelvin Grove, 4059 Australia; 3 Department of Obstetrics and Gynaecology, Royal Brisbane and Women’s Hospital, Herston, 4006 Australia; 4 Department of Obstetrics and Gynaecology, Townsville University Hospital, Douglas, 4814 Australia; 5 Department of Urology, Mater Hospital, South Brisbane, 4101 Australia; 6 Department of Urology, Royal Brisbane and Women’s Hospital, Herston, 4006 Australia; 7 Department of Obstetrics and Gynaecology, Redcliffe Hospital, Redcliff, 4020 Australia

**Keywords:** Urinoma, Rupture, Spontaneous, Pregnancy, Hydronephrosis

## Abstract

**Background:**

Spontaneous renal fornix rupture (SRFR) causing urinoma is an uncommon but serious condition in pregnancy. Limited information is available to describe the natural history and outcomes to guide appropriate treatment. The aim of this study was to determine the natural history and outcomes of SRFR to determine appropriate management recommendations.

**Methods:**

A systematic review of literature databases was performed, using the Meta-analysis Of Observational Studies in Epidemiology (MOOSE) checklist from 1950 – April 2020. Inclusion criteria included any urinary extravasation from the kidney or ureter during pregnancy, or in the 8 weeks following delivery, confirmed via imaging or surgery. Haematomas and non-confirmed cases were excluded.

**Results:**

A total of 1579 records were originally identified, of which 39 case reports were appropriate for inclusion. SRFR was most commonly reported during the first pregnancy (72%), 19/30 during the third trimester and 9 in the post-natal period. All patients presented with pain, with haematuria positive on urine dipstick in only 36% of 26 reported cases. Ultrasound was the most frequently used imaging modality, resulting in a diagnosis in 42% of cases. All cases reported on treatment procedures including ureteric stents (46%), percutaneous drain (15%), conservative management (15%), nephrostomy (13%) and ureteral catherization (10%). Long term urological outcomes were positive, however women suffering SRFR were significantly more likely to undergo pre-term labour.

**Conclusion:**

While selected cases may be successfully managed conservatively, urinary diversion, through ureteric stents, should be considered the management of choice in these individuals. Clinicians should be mindful of an increased risk of premature delivery and its’ associated negative fetal outcomes.

## Background

Ureteric outflow obstruction may occur anywhere between the kidney and vesico-ureteric junction, resulting in increased intraluminal or renal pelvis pressure. The renal fornix is the most common site for spontaneous perforation or rupture, followed by the upper ureter [1]. Spontaneous renal fornix rupture (SRFR) is often encountered in urological practice due to the presence of ureteral calculi, extrinsic compression from malignancy or mass, and instrumentation [2, 3], but is otherwise uncommon in their absence [4]. The consequences of urine extravasation into the surrounding perinephric tissue include formation of a urinoma, abscess, urosepsis and renal impairment.

Increased intraluminal pressure is commonly observed with physiologic hydronephrosis of pregnancy due to both mechanical and hormonal stimuli [5]. Whilst flank pain and pyelonephritis secondary to over distension are common manifestations of this physiologic phenomenon, rupture of the renal collecting system may rarely occur, especially with excessive intraluminal pressures [1].

Due to the paucity of published data for this condition, clinical confidence is limited in identifying at-risk patients, further complicated by vague, non-specific findings on clinical assessment and laboratory testing along with limitations in use of appropriate diagnostic imaging. Pregnancy adds further complexity, with possible fetal and uterine causes of pain. Pre-term labour, uterine irritability, and placental abruption [6–8] have all been confused for calyceal rupture, resulting in potentially detrimental interventions for both the mother and fetus. Urgent urological assessment is required when SRFR or significant obstruction is suspected, although optimal diagnostic pathways and management are currently unknown.

Whilst urologic long-term prognosis is favourable in cases of physiological obstruction, the effects of SRFR on pregnancy outcomes are not well known. Although rare, the potential for harm in these cases is amplified for both mother and fetus, making early diagnosis and appropriate management crucial. Consequently, the aim of this study was to identify risk factors and common clinical presentations, diagnostic pathways, management options and patient outcomes for SRFR in pregnancy.

## Methods

A systematic review of the published literature was conducted using the Meta-analysis Of Observational Studies in Epidemiology (MOOSE) checklist [9] (Supplement 3). The search was conducted using journal databases including PubMed, Embase, Cochrane Library, Scopus and Web of Science. The search terms included “Kidney”, “ureter” or “kidney pelvis” and “calyceal”, “fornix”, “perirenal” or “perinephric” and “rupture”, “extravasation” or “urinoma” and “pregnancy” or “maternal” (see supplement 2 for specific search terms used). Cross-references were identified and reviewed for additional suitable publications.

### Inclusion/exclusion criteria

All published literature was considered, including peer reviewed journal articles, case reports and case series. Inclusion criteria included spontaneous urine extravasation from a non-traumatic event, from the renal pelvis or ureters and in a pregnant patient (including up to 8 weeks post-partum) where diagnosis was made as a result of imaging or during surgical exploration. Exclusion criteria included articles not available in English, renal haematomas or, urinary extravasation not confirmed on either imaging or surgical exploration, unpublished cases and abstracts. Suitability was assessed by two independent researchers (SS and AG) using the JBI Critical Appraisal checklist for Case Reports [10], with a 3rd person (MM) available to resolve any conflicting opinions.

### Outcome measures

Primary outcomes that were assessed included patient symptoms and signs at presentation, imaging modalities used for diagnosis and outcomes of treatment in relation to regression of disease and complications during pregnancy. Secondary outcomes included risk factors, both obstetric and urologic, and biochemical and imaging investigation findings.

### Data management

Data was extracted from the identified case reports into Microsoft Excel and collated for assessment (supplement 2). Statistical analysis was completed using SPSS Version 25 (IBM Corp, Armonk, NY, USA). Categorical variables were summarised as frequency and percentage and continuous variables as mean and standard deviation (SD) or median (interquartile range (IQR)). Statistical analysis including Kaplan-Meier survival analysis was performed using MedCalc for Windows, Version 18 (MedCalc Software; Ostend, Belgium).

## Results

### Literature search

**Fig. 1:**
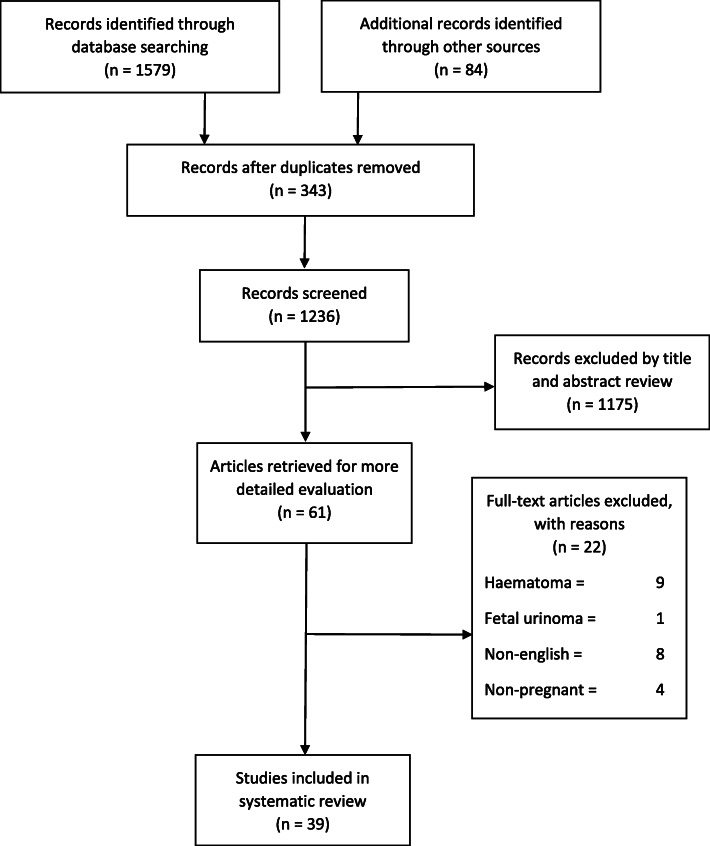
MOOSE systematic review flow chart

Following a review of 1579 database entries and removal of 343 duplicates and other entries (1175 due to not meeting exclusion criteria), 39 articles were eligible for final inclusion in the analysis (Fig. 1). All of these articles were published case reports from peer-reviewed journals and are available in supplementary Table 1. No case series or larger population studies were identified. The earliest reported case satisfying the study requirements was reported in 1968 [11], with eight cases published between 1980 and 2000, 11 cases between 2000 and 2010, and 16 cases identified from the last decade. A total of 18 cases were excluded including those renal ruptures that resulted in haematoma formation [9], publication in a language other than English [8] and fetal urinoma [1].

### Patient demographics

The mean age of the patients included in the review was 26.7 (SD 5.5) years. Of these patients, 23/32 (72%) were in their first pregnancy. SRFR most commonly occurred in the antenatal period in 30/39 (77%) and most frequently in the third trimester 19/30 (63%), with only one rupture reported in the first trimester [12]. Nine patients suffered SRFR in the post-partum period with a mean time to diagnosis 17 days and range from 1 day to 5 weeks. Table 1 provides the patient demographics, gestation at diagnosis and delivery, parity and method of delivery.

**Table 1:** Characteristics of patients included in the review

Characteristic	Antenatal diagnosis (*n* = 30) *n* (%)	Postnatal diagnosis (*n* = 9) *n* (%)	Overall (*n* = 39) *n* (%)
Age – Mean (SD) *n* = 39	26.7 (5.5)	26.2 (3.1)	26.6 (5.1)
Gestation at diagnosis – Mean (SD), Range	28 (8), (5–40)	17.8 days, (1 day – 5 weeks)	n/a
Gestation at Delivery – Median (IQR) *n* = 32	37 (34, 40)^^^	40 (38, 41)^*^	37 (34, 40)
Parity (*n* = 32)			
Primigravid	21 (75%)	2 (50.0%)	23 (71.9%)
Multigravid	7 (25%)	2 (50.0%)	9 (28.1%)
Delivery (*n* = 32)			
Vaginal/Instrumental	19 (70.4%)	3 (60.0%)	22 (68.8%)
Caesarean	8 (29.6%)	2 (40.0%)	10 (31.3%)

^^^*n* = 28, ^*^ n-4, *n/a* not available

### Urologic history

The clinical information was generally not well reported in the literature, with a history of renal calculi (14 reports, 2 positive) and previous urinary tract infection (23 reports, 3 positive) being the most common. None of the publications mentioned a history of underlying chronic renal disease, previous renal tract surgery or pre-existing medical conditions. Table 2 delineates the clinical presentation, imaging findings and the treatment modlaities used in patients included in the review.

**Table 2:** Clinical presentation, imaging and treatment of patients included in the review

Characteristic	Diagnostic criteria, *n* (%)
Symptoms	
*Flank pain (*n* = 39)	39 (100%)
Haematuria (*n* = 28)	10 (35.7%)
Fever (*n* = 20)	8 (40%)
Dysuria (*n* = 12)	2 (16.7%)
*Nausea/vomiting (*n* = 39)	12 (38.7%)
Previous medical history	
Renal calculi (*n* = 13)	2 (15.4%)
White cell count	
Elevated (*n* = 21)	11 (52.4%)
Diagnostic imaging findings (*n* = 39)	
Dilated kidney/urinary tract	22 (56.4%)
Urinoma or perinephric/pelvic collection	15 (38.4%)
Presence of calculi	2 (5.1%)
Treatment (*n* = 39)	
Stent	18 (46.1%)
Ureteric catheter	4 (10.3%)
Percutaneous drain	5 (15.4%)
Nephrectomy	1 (2.6%)
Nephrostomy	5 (12.8%)
Conservative	6 (15.4%)

*n* number of patients where data is available.

*Pain and Nausea and vomiting were interpreted as yes or no

4 patients had laparotomy in addition to above treatment.

### Clinical presentation

All patients presented with pain, with 14 of these (36%) describing it as severe. Pain preceded the clinical presentation from 1 day to 6 weeks. The right side was most frequently affected (67%, 26/39), with two patients (5%) complaining of bilateral discomfort. Pain was described in the flank or abdomen (23%, 9/39) and two patients (5%) presented with peritonism. The other symptoms associated with SRFR are shown in Table 2.

Urine microscopy was reported in 26 of 39 cases, with only 10 (36%) reporting haematuria. Eight cases were positive for urinary leukocytes (31%), with seven of these reporting a positive urine culture (27%%). Blood chemistry revealed elevated white cell counts in 52% of the 21 reported cases and a raised C-reactive protein was present in 3 out of 10 reported cases. Blood creatinine was known to be elevated in 19% of cases where it was reported.

### Imaging and diagnosis

**Fig. 2:**
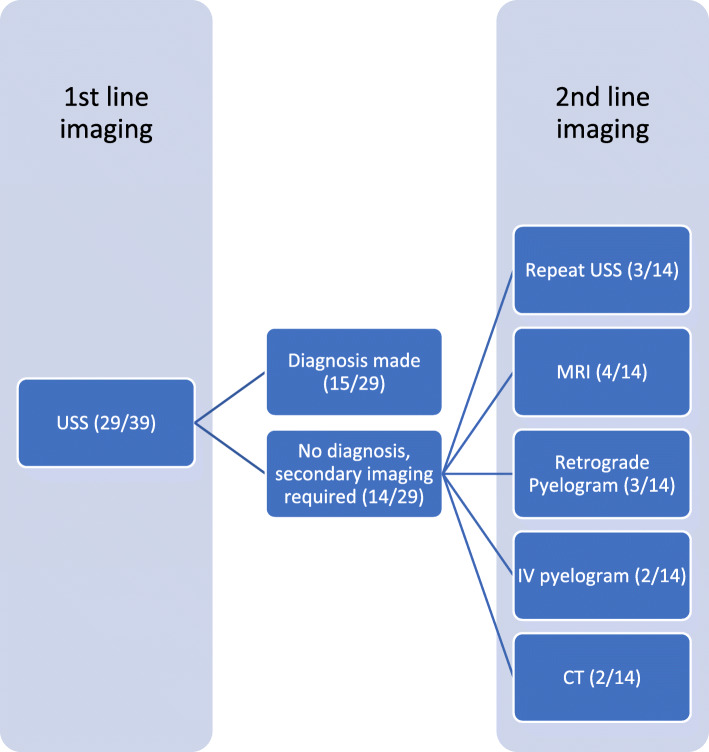
Imaging of choice for successful diagnosis of SRFR

Urinary extravasation was confirmed on imaging studies in all patients, as shown in Table 2. Ultrasound was the most common investigation performed and was used in 29 cases (74%) with a diagnosis of urinary extravasation made in 15 of these (52%). When SRFR was not identified on USS in the first instance, a second line diagnostic imaging modality was utilized as shown in Fig. 2. The earliest successful use of USS for diagnosis of SRFR was by Maresca and colleagues in 1981 [13], with the three cases prior to this utilising IV pyelograms for diagnosis. In those cases where USS was available and not performed, four cases were diagnosed with CT and three with MRI.

**Fig. 3:**
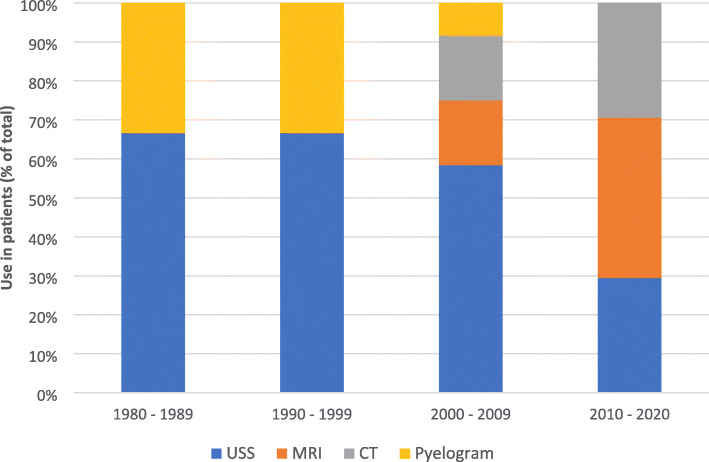
Evolution of Diagnostic imaging modalities used to diagnose SRFR

Preferences for imaging choice in this clinical scenario evolved over time (Fig. 3), with an increased popularity and role of MRI observed, being used in 8/12 (67%) of cases between 2015 and 2020, when compared to only 3/27 (11%) before 2015.

CT was the modality of choice for patients who presented post-partum, used in 7/8 (88%) of studies. Three of these studies [14–16] used CT to confirm positive USS findings, while the remainder of studies used CT as a primary diagnostic tool. One case used CT as their primary imaging modality during the antepartum period [17].

The majority of SRFR (79%) were reported as being secondary to, or associated with, pregnancy. One author suggested breech presentation of the fetus as an unlikely primary cause, suggesting an increased pressure on the right ureter at the pelvic brim [18]. Other reported primary causes contributing to the rupture included ureteric calculi (3/39), anatomical defect (2/39), intra-venous fluid bolus with calculi (1/39), complication from caesarean section (1/39) and pre-eclampsia (1/39).

### Management

**Fig. 4:**
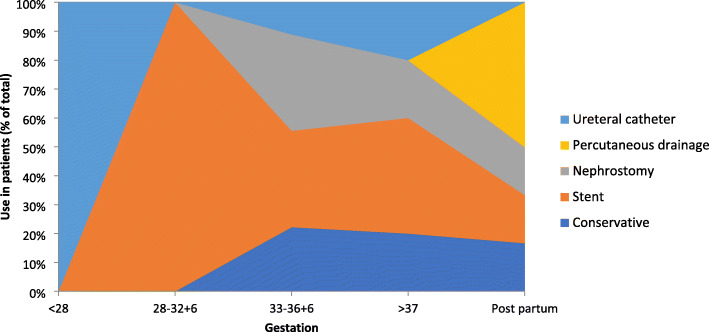
Treatments utilised depending on gestation at diagnosis

Women were commonly treated with ureteric stenting, especially at earlier gestations (Fig. 4) (46%, 18/39), with JJ stents used in 10 cases, one “double pigtail”, and five undescribed stents. All stents were removed between two- and eight-weeks post-partum (mean: 4 weeks), with a mean in-situ time of 8.8 weeks (range 2–21 weeks). Nephrostomy was performed in five cases (13%), with five cases each of ureteral catheterisation and percutaneous peri-nephric drainage. Conservative management was performed in 6/39 cases (15%), utilising antibiotics and analgesia, commonly used in patients reaching full-term gestation (Fig. 4). Cohen and Pearlman described the only nephrectomy that was performed, in 1968, due to a markedly hydronephrotic kidney and ureteropelvic obstruction found during exploratory laparotomy [11].

### Outcomes and complications

Overall, no significant long-term urologic complications were reported, with all patients responding well to treatment. Appendicectomy was performed in six cases following an incorrect original differential diagnosis and in four of these instances via a laparotomy. One patient required haemodialysis during the acute phase, however long-term recovery for this specific patient was not reported [12].

**Fig. 5:**
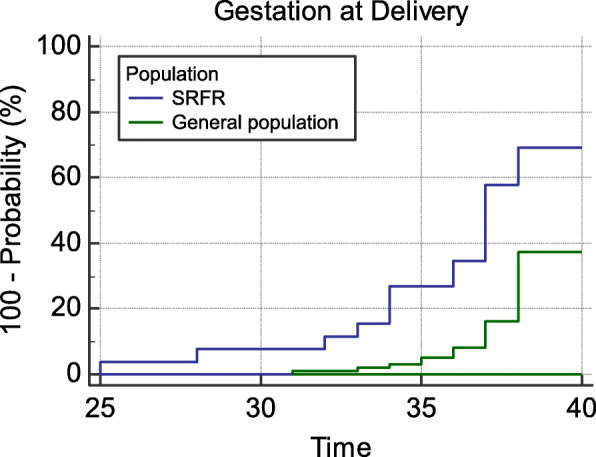
Comparative Kaplan-Meier probability of delivery according to gestation

Pregnancy complications were more common, with patients (n = 32) delivering at an average gestation of 36.3 weeks (IQR: 34.8–40). Eleven patients (34%) delivered pre-term with three of these delivering at a gestation < 32 weeks. Pregnancies went to term in 21 cases (66%). When Kaplan-Meier survival analysis was applied to this scenario, with the endpoint being gestation time at delivery censored at 37 weeks, patients with SRFR (36.3, 95% CI 35–37.9 weeks) were significantly more likely to undergo preterm labour than the general population (19, 38.4, 95% CI 37.9–38.8 weeks; p = 0.0001; Fig. 5). Only one patient requested, and was granted, pre-term delivery at 35 weeks secondary to pain from SRFR [19].

Two-thirds of the patients delivered vaginally or instrumentally (69%, 22/32), and the remaining by caesarean section (31%, 10/32). One patient developed a perinephric haematoma following a double-J stent insertion which resulted in a spontaneous miscarriage at 19 weeks gestation [20]. A single neo-natal death occurred most likely due to extreme prematurity, following a 26-week delivery in a case of suspected cervical incompetence [7].

## Discussion

We present, to our knowledge, the first systematic review conducted to assess the diagnosis, management and outcomes relating to SRFR in pregnancy. The analysis suggests that urological outcomes are generally excellent, with the only reported long term morbidity occurring after a nephrectomy in 1968 [11]. Fetal outcomes however, may be adversely affected by the increased rate of premature delivery found in this population, a finding not reported previously. The management and outcomes for obstetric population have only been published in single case studies, with results from larger retrospective cohort studies in the non-pregnant population extrapolated to pregnant women. Gershman et.al [21], the largest study to date, retrospectively analysed 108 cases of renal pelvis rupture, and found only two cases relating to pregnancy. All causes of extrinsic ureteric compression contributed to a total of 14% of cases, with ureteric calculi accounting for the vast majority (74%). Therefore, extrapolation of these findings to the pregnant population, in order to make evidence-based decisions, may be precarious. While there are recommendations available on appropriate management for urinary tract obstruction due to calculi in pregnancy [22–24], these do not include SRFR. This study offers unique insights for the treating clinician into the outcomes of these patients and the treatment modalities that have been previously used successfully.

Pregnancy outcomes for patients with SRFR have only been described in case reports. The analysis of the data suggests that delivery occurred earlier in these patients at a mean gestation of 36.3 weeks. Pre-term delivery, defined as < 37 completed weeks gestation, occurred in 34% (11/32) of the reported cases compared to a background prevalence of 8.4% in Australia [25] (Fig. 5). Rates of neonatal complications, including respiratory distress, hypoglycaemia, hypoxic ischaemic encephalopathy and intraventricular haemorrhage, are inversely proportional to gestational age at delivery [26]. To a lesser extent, fetal outcomes are known to be adversely affected between 37 and 39 weeks, compared to those babies born at or beyond 39 weeks [27], as found in 63% of our reported cases. Although limited interpretations can be made due to scant data reported over multiple decades and at differing geographical locations, these numbers appear concerning. Importantly however, it does not appear that pre-term delivery for the exclusive reason of SRFR has been advocated in the published studies.

Further extrapolation of specific neonatal outcomes is difficult due to the under-reporting noted in the case reports, with adequate information available in only 4 cases. Although the numbers of caesarean sections vary widely across different centres, the rate does not appear to be increased in these patients (32% when compared to the developed world) [25]. Besides a trend towards earlier delivery gestation, there were no significant increases in rates of other antenatal or delivery complications. As such, patients with SRFR should be delivered for standard obstetric indications only. In patients at a higher risk of preterm birth, outcomes may be improved with the early use of corticosteroid injections in order to accelerate fetal lung maturity [26].

**Fig. 6:**
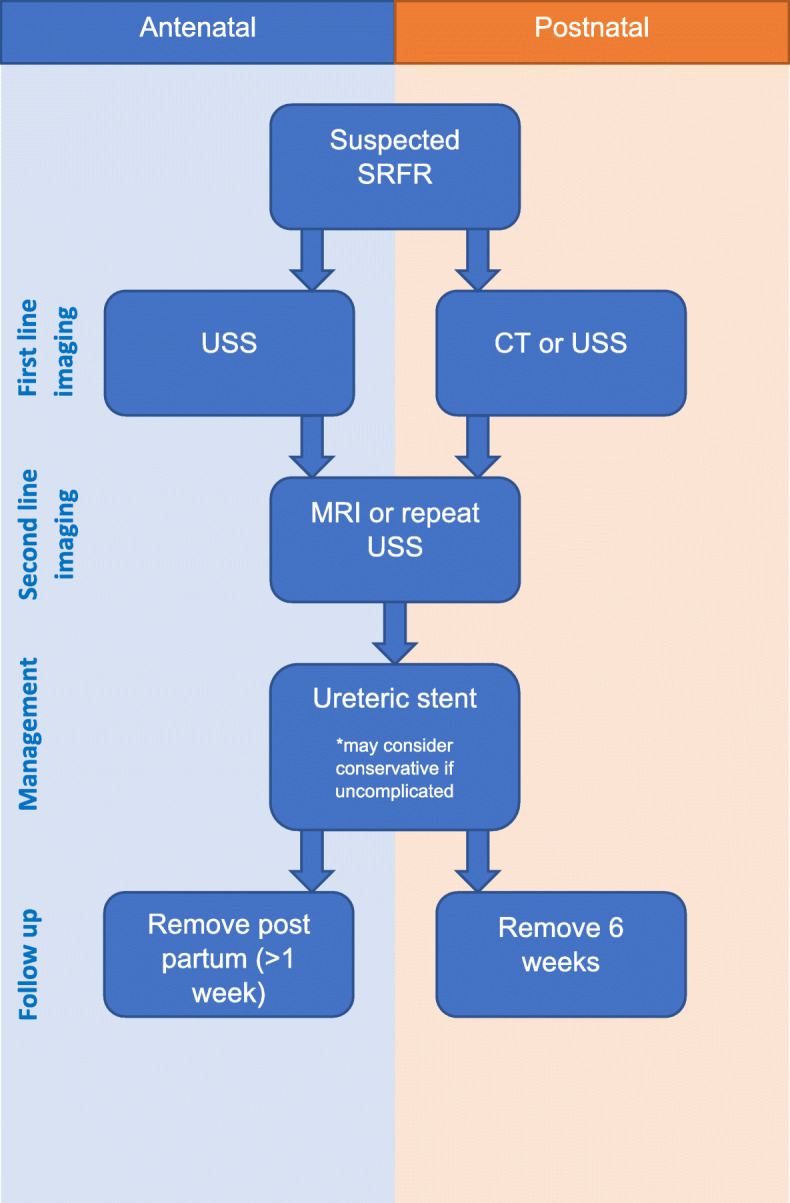
Flow chart for the diagnosis and management of SRFR related to pregnancy

Reassuringly, there were no adverse urological outcomes reported in any of the patients and all women made a complete recovery regardless of treatment used [11]. A suggested management pathway is shown in Fig. 6. Urinary diversion, most commonly through ureteric stenting, nephrostomy and ureteric catheterisation, was used in most cases and is recommended by the authors as first line management. In all cases where ureteric stenting was performed, removal occurred in the post-partum period (mean: 4 weeks), a decision supported by increased anaesthetic risks during the ante-partum, and early post-partum period [28]. Conservative management may also be considered as patients approach full-term in the pregnancy, especially in those that are otherwise uncomplicated and require minimal analgesia [24]. Unfortunately, it may not always be successful and urinary diversion may be required. Boekhorst describe a case in which conservative management failed due to ongoing high analgesic requirements and eventually required stent insertion, resulting in an almost immediate resolution of pain [29]. On anlaysis of the data included in this review, the most common cause of harm occurred as a result of surgical intervention following an incorrect initial diagnosis. Appendicectomy was performed inappropriately in 17% of the instances, with the most recent case reported in 2016 [6]. Similar findings have been discussed in non-pregnant patients with aberrant diagnosis in up to 50% cases [30].

In this review, ultrasound successfully diagnosed urinary extravasation from the renal pelvis in more than half of the cases. Hence, ultrasound diagnosis should be considered as the first line investigation for any patient presenting with flank pain in pregnancy. Even in those cases where diagnosis was not made in the first instance, a repeat ultrasound may be successful (Fig. 2). USS has the added benefit of concurrent diagnosis of hydronephrosis, presence of calculi and investigation of fetal well-being, all of which aid the clinician in patient care. In those cases where ultrasound has been unable to diagnose urinary extravasation on the initial scan, MRI is recommended. It has been successfully used to diagnosis SRFR 75% of cases reported in the last 5 years (Fig. 5). In low resource settings, where MRI may not be readily available, other modalities such as low-dose retrograde pyelograms may be appropriate as the radiation level is below the suggested maximum according to the American College of Obstetricians and Gynaecologists [31]. Understandably, due to improvements in imaging options, retrograde pyelography has not been widely used since the 1990s. Computed tomography scanning, even with low dose protocols, should be avoided in pregnant patients due to the potential risks to the fetus [31], and the desire to avoid ionising radiation [32]. For those women in the post-partum period, a CT scan may be of value, however radiation guarding of the lactating breasts is important.

There are several limitations of this study. The heterogeneous nature of the published data resulted in correlation of the risk factors and analysis of findings difficult. Additionally, inconsistent reporting is likely to result in a bias of causative association estimates, and thus high-powered statistical analysis of the pooled data was not feasible. Furthermore, with changes in urologic practice over the past 30 years, the heterogeneity of management decisions across this time frame also limits the strength of recommendations. Standard clinical management of patients presenting with SRFR in pregnancy is currently highly variable and treatment of these patients is based largely on extrapolation from studies in the non-pregnant population. Until now, patient outcomes have only been reported on single cases. Reassuringly, this review demonstrates that management has been reasonably effective to date with positive urological and pregnancy outcomes, regardless of the treatments used.

## Conclusion

Outcomes for patients with SRFR in pregnancy are generally positive. While selected cases may be successfully managed conservatively, urinary diversion, through ureteric stents, should be considered the management of choice in these individuals. This review demonstrates an a trend towards an increase in the risk of premature delivery in this patient group warranting awareness by the treating obstetrician. Additionally, delivery is recommended only for standard obstetric indications. The strength of these recommendations is low due to the small number of reported cases and further research is in this field is suggested to improve patient outcomes.

## Supplementary information

**Additional file 1.**

**Additional file 2.**

**Additional file 3.** MOOSE (Meta-analyses Of Observational Studies in Epidemiology) Checklist.

## Data Availability

The datasets used and/or analysed during the current study are available from the corresponding author on reasonable request.

## Abbreviations

SRFR: Spontaneous renal fornix rupture
CT: Computed tomography
MRI: Magnetic resonance imaging
USS: Ultrasound scan
