# Supplementary material for: Spontaneous renal fornix rupture in pregnancy and the post partum period: a systematic review of outcomes and management
Source: BMC Urol. 2020 Aug 4;20:116. doi: 10.1186/s12894-020-00660-z (PMC7405429; doi:10.1186/s12894-020-00660-z)
Supplement: Supplementary file 1 — Additional file 1. [file 12894_2020_660_MOESM1_ESM.docx]

| Publication | Year | Parity* | Gestation diagnosis | | Gestation dELIVERY | | AgE | Presentation | | Symptoms | | Haematuria | | Fever | | Dysuria | | | WCC | |  | |
| --- | --- | --- | --- | --- | --- | --- | --- | --- | --- | --- | --- | --- | --- | --- | --- | --- | --- | --- | --- | --- | --- | --- |
| Cohen (1) | 1968 | Multiparous | 37 | 38 | | 35 | | | pain | | Nausea/vomiting | | yes | | -/- | | -/- | -/- | |  | |  |
| Middleton (2) | 1980 | Multiparous | 18 | 40 | | 26 | | | acute right flank and RUQ pain | | Nausea/vomiting | | yes | | -/- | | -/- | -/- | |  | |  |
| Noe (3) | 1980 | Nulliparous | 40 | 40 | | 20 | | | flank pain and Right upper quadrant pain | | Fever | | no | | -/- | | -/- | -/- | |  | |  |
| Eaton (4) | 1981 | Nulliparous | 30 | 37 | | 27 | | | bilateral flank pain | | Nausea/vomiting | | -/- | | yes | | -/- | elevated | |  | |  |
| Maresca (5) | 1981 | Multiparous | 18 | 40 | | 34 | | | right flank pain, severe | | Nausea/vomiting | | no | | -/- | | -/- | normal | |  | |  |
| Kramer (6) | 1983 | Nulliparous | 30 | 40 | | 18 | | | right flank pain, severe | | Nausea/vomiting | | no | | -/- | | -/- | normal | |  | |  |
| Dhabuwala (7) | 1984 |  | 28 | 40 | | 19 | | | right flank pain | | Nausea/vomiting | | no | | yes | | -/- | elevated | |  | |  |
| Meyers (8) | 1985 | Nulliparous | 27 | 28 | | 18 | | | right flank pain and right lower abdominal pain with peritonism, severe | |  | | yes | | no | | no | -/- | |  | |  |
| Oesterling (9) | 1988 | Nulliparous | 20 | 40 | | 36 | | | right flank pain, Right lower abdominal pain | | Nausea/vomiting | | -/- | | -/- | | -/- | -/- | |  | |  |
| Van Winter (10) | 1991 | Nulliparous | 34 | 37 | | 20 | | | right flank pain | | Nausea/vomiting | | no | | -/- | | -/- | normal | |  | |  |
| Hamoud (11) | 1994 | Nulliparous | 37 | 37 | | 29 | | | right flank pain, lower abdominal pain with peritonism | |  | | -/- | | no | | no | normal | |  | |  |
| Royburt (12) | 1994 | Multiparous | 33 | 36 | | 29 | | | left flank pain | |  | | yes | | no | | no | elevated | |  | |  |
| Hwang (13) | 2000 | Multiparous | 28 | -/- | | 26 | | | right flank pain, severe | |  | | no | | -/- | | -/- | -/- | |  | |  |
| Nabi (14) | 2001 | Unknown | 18 | 19 | | 28 | | | right flank pain | |  | |  | | -/- | | -/- | normal | |  | |  |
| Satoh (15) | 2002 | Nulliparous | 20 | 41 | | 34 | | | right flank pain | |  | | no | | -/- | | -/- | normal | |  | |  |
| Jang (16) | 2006 | Unknown | 4w PP |  | | 31 | | | right flank pain, severe | |  | | no | | -/- | | -/- | -/- | |  | |  |
| Lang (17) | 2006 | Multiparous | -/- | -/- | | 27 | | | bilateral flank pain | |  | | yes | | yes | | yes | elevated | |  | |  |
| Lo (18) | 2007 | Nulliparous | 32 | 32 | | 33 | | | left flank pain | |  | | no | |  | | -/- | normal | |  | |  |
| Ushioda (19) | 2008 | Nulliparous | 21 | 39 | | 27 | | | left flank pain, lower abdominal pain | |  | | no | | no | | no | normal | |  | |  |
| Sidra (20) | 2009 | Nulliparous | 34 | 34 | | 28 | | | right flank pain | |  | | no | | no | | -/- | -/- | |  | |  |
| Huang (21) | 2009 | Nulliparous | 19 | 25 | | 27 | | | right flank pain, severe | |  | | -/- | |  | | -/- | -/- | |  | |  |
| Machado (22) | 2009 | Nulliparous | 31 | 34 | | 20 | | | right flank pain, severe | |  | | -/- | | yes | | no | -/- | |  | |  |
| Tang (23) | 2009 | Nulliparous | D5 PP | 37 | | 31 | | | right flank pain | |  | | yes | | -/- | | -/- | -/- | |  | |  |
| Matsubara (24) | 2010 | Nulliparous | 34 | 34 | | 38 | | | left flank pain, back pain | |  | | yes | | -/- | | -/- | -/- | |  | |  |
| Efrimescu (25) | 2013 | Nulliparous | 28 | 36 | | 31 | | | left flank pain, severe | |  | | yes | | no | | no | elevated | |  | |  |
| Khandelwal (26) | 2013 | Unknown | 5w PP | - | | 25 | | | left flank pain, severe | |  | | no | | -/- | | -/- | -/- | |  | |  |
| Jalbani (27) | 2014 | Unknown | 5 | - | | 22 | | | right sided abdominal pain | | Nausea/vomiting | | -/- | | yes | | yes | -/- | |  | |  |
| Kumar (28) | 2014 | Unknown | 5w PP | - | | 25 | | | right flank pain | |  | | no | | -/- | | -/- | -/- | |  | |  |
| Upputalla (29) | 2014 | Nulliparous | 23 | 37 | | 26 | | | right sided abdominal pain | |  | | -/- | | no | | no | normal | |  | |  |
| Boekhorst (30) | 2015 | Multiparous | 34 | 37 | | 27 | | | right sided abdominal pain | | Nausea/vomiting | | no | | no | | no | elevated | |  | |  |
| Hanson (31) | 2015 | Nulliparous | 33 | 33 | | 29 | | | right flank pain | | Nausea/vomiting | | no | | no | | -/- | elevated | |  | |  |
| Narashimhulu (32) | 2015 | Nulliparous | PP | - | | 22 | | | left flank pain and contraction pain | |  | | yes | | no | | no | elevated | |  | |  |
| Patel (33) | 2015 | Nulliparous | 38 | 38 | | 24 | | | right flank pain | | Nausea/vomiting | | no | | -/- | | -/- |  | |  | |  |
| Fluke (34) | 2016 | Nulliparous | 30 | 38 | | 21 | | | left flank pain | |  | | -/- | | -/- | | -/- | elevated | |  | |  |
| Deshmukh (35) | 2016 | Unknown | 3w PP | 40 | | 26 | | | right sided abdominal pain | |  | | -/- | | -/- | | -/- | -/- | |  | |  |
| Traveiso (36) | 2018 | Nulliparous | 22 | 37 | | 25 | | | right sided flank pain | |  | | no | | no | | no | normal | |  | |  |
| Chen (37) | 2019 | Nulliparous | 26 | 35 | | 23 | | | left flank pain | |  | | yes | | no | | -/- | -/- | |  | |  |
| Verhaeghe (38) | 2019 | Multiparous | D1 PP | 41 | | 26 | | | left abdominal and flank pain | |  | | -/- | | yes | | -/- | elevated | |  | |  |
| Rickard-Bell (39) | 2020 | Multiparous | D1 PP | 40 | | 23 | | | right flank pain | |  | | no | | yes | | -/- | elevated | |  | |  |
|  |  |  |  |  | |  | | |  | |  | |  | |  | |  |  | |  | |  |

| Publication | Previous claculi | prior renal disease | Initial Diagnosis via Ultrasound | Additional Imaging | | treatment | | Delivery Method | |  |
| --- | --- | --- | --- | --- | --- | --- | --- | --- | --- | --- |
| Cohen (1) | -/- | -/- | Not performed | | Retrograde pyelogram | | Nephrectomy, Laparotomy | | Vaginal delivery | |
| Middleton (2) | no |  | ground glass appearance of right kidney | | Retrograde pyelogram | | Nephrostomy | | Vaginal delivery | |
| Noe (3) | no | no | -/- | | -/- | | Conservative | | Caesarean section | |
| Eaton (4) |  |  | Results not reported | |  | | Nephrostomy | | Instrumental delivery | |
| Maresca (5) | -/- | -/- | -/- | | Retrograde pyelogram | | ureteric catheter | | Vaginal delivery | |
| Kramer (6) | -/- | -/- | hydronephrosis, urinoma | | Retrograde pyelogram | | Conservative | | Vaginal delivery | |
| Dhabuwala (7) | -/- | -/- | right hydronephrosis, urinoma | | Retrograde pyelogram | | Ureteric stent, Laparotomy | | Vaginal delivery | |
| Meyers (8) | no | -/- | retroperitoneal collection of fluid around right kidney accompanied by calicectasis | | Retrograde pyelogram | | Ureteric Stent | | -/- | |
| Oesterling (9) | -/- |  | hydronephrosis, urinoma | | Retrograde pyelogram | | ureteric catheter | | Vaginal delivery | |
| Van Winter (10) | -/- | -/- | hydronephrosis, urinoma | | Retrograde pyelogram | | Ureteric Stent | | Vaginal delivery | |
| Hamoud (11) | no | no | hydronephrosis | | -/- | | ureteric catheter | | Caesarean section | |
| Royburt (12) | -/- | -/- | bilateral mild hydroureter L>R, L perirenal urinoma and urine extravasation | | -/- | | Conservative | | Vaginal delivery | |
| Hwang (13) | -/- | -/- | right hydronephrosis and hydroureter with perinephric fluid with extravasation of urine | | -/- | | ureteric catheter | | -/- | |
| Nabi (14) | -/- | -/- | mild dilatation of right calices, renal pelvis, and proximal ureter (consistent with pregnancy) | | -/- | | Ureteric stent | | Spontaneous miscarriage (19 weeks) | |
| Satoh (15) | -/- | -/- | hydronephrotic right kidney with small preinephric collection. hydronephrotic left kidney: non-functioning | | Retrograde pyelogram | | Ureteric stent | | Caesarean section | |
| Jang (16) | no | no | mild hydronephrosis | | CT | | Percutaneous Drain | | Caesarean section | |
| Lang (17) | no | no | mild hydronephrosis | | CT | | Percutaneous Drain | | Caesarean section | |
| Lo (18) | -/- | -/- | huge echo-poor collections around both kidneys | | CT | | Percutaneous Drain | | Caesarean section | |
| Ushioda (19) | -/- | -/- | -/- | | CT | | Ureteric stent | | Caesarean section | |
| Sidra (20) | no | no | hydronephrosis bilateral, hydroureter (performed after delivery) | | -/- | | Ureteric stent | | Vaginal delivery | |
| Huang (21) | -/- | -/- | mild bilateral hydronephrosis | | Retrograde pyelogram | | Ureteric Stent | | Vaginal delivery | |
| Machado (22) | -/- | -/- | hydronephrosis, hydroureter | | -/- | | Ureteric Stent | | -/- | |
| Tang (23) | -/- | -/- | right perinephric fluid collection | | -/- | | Ureteric stent | | -/- | |
| Matsubara (24) | no | no | IUGR, mild right hydronephrosis | | Retrograde pyelogram | | Ureteric stent | | Caesarean section | |
| Efrimescu (25) | no | no | left renal hydronephrosis | | -/- | | Ureteric stent | | Caesarean section | |
| Khandelwal (26) | -/- | -/- | bilateral hydronephrosis | | -/- | | Percutaneous drain | | Instrumental delivery | |
| Jalbani (27) | no | no | bilateral dilatation of renal collecting system | | CT | | Percutaneous Drain | | Vaginal delivery | |
| Kumar (28) | yes | -/- | echo-poor collection around the kidneys | | -/- | | Ureteric stent | | -/- | |
| Upputalla (29) | no | no | large perinephric collection with multiple stones in the renal pelvis | | CT | | Percutaneous Drain | | Vaginal delivery | |
| Boekhorst (30) | -/- | -/- | echo-poor collection around the kidneys | | MRI | | Nephrostomy | | Vaginal delivery | |
| Hanson (31) | -/- | -/- | -/- | | MRI | | Ureteric stent | | Vaginal delivery | |
| Narashimhulu (32) | yes | -/- | slight bilateral dilation of upper urinary tract. appendix not seen. no kidney/gallbladder stones | | MRI | | Conservative | | Vaginal delivery | |
| Patel (33) | no | no | mild left pelviectasis not out of proportion with normal pregnancy, small area of echogenicity ?stone | | CT / Retrograde pyelogram | | Ureteric stent | | Vaginal delivery | |
| Fluke (34) | no | no | normal | | CT | | Conservative | | Caesarean section | |
| Deshmukh (35) | -/- | -/- | -/- | | Retrograde pyelogram | | Ureteric Stent | | -/- | |
| Traveiso (36) | -/- | -/- | mild right hydronephrosis | | CT | | Conservative | | Vaginal delivery | |
| Chen (37) | -/- | -/- | Right ureteric tapering | | MRI only | | Nephrostomy | | Vaginal delivery | |
| Verhaeghe (38) | -/- | -/- | Hydronephrosis and surrounding cyst | | MRI | | Nephrostomy | | Caesarean section | |
| Rickard-Bell (39) | -/- | -/- | -/- | | CT only | | Ureteric stent | | Instrumental delivery | |

**References**

1. Cohen SG, Pearlman CK. Spontaneous rupture of the kidney in pregnancy. The Journal of urology. 1968;100(4):365-9.
2. Middleton AW, Jr., Middleton GW, Dean LK. Spontaneous renal rupture in pregnancy. Urology. 1980;15(1):60-3.
3. Noe HN, Raghavaiah NV. Spontaneous peripelvic extravasation of urine during pregnancy. South Med J. 1980;73(6):809-10.
4. Eaton A, Martin PC. Ruptured ureter in pregnancy--a unique case? Br J Urol. 1981;53(1):78-9.
5. Maresca L, Koucky CJ. Spontaneous rupture of the renal pelvis during pregnancy presenting as acute abdomen. Obstetrics and Gynecology. 1981;58(6):745-7.
6. Kramer RL. Urinoma in pregnancy. Obstetrics and Gynecology. 1983;62(3 Suppl.):26S-8S.
7. Dhabuwala CB, Riehle RA. SPONTANEOUS RUPTURE OF A HYDRONEPHROTIC KIDNEY DURING PREGNANCY. Urology. 1984;24(6):591-4.
8. Meyers SJ, Lee RV, Munschauer RW. Dilatation and nontraumatic rupture of the urinary tract during pregnancy: A review. Obstetrics and Gynecology. 1985;66(6):809-15.
9. Oesterling JE, Besinger RE, Brendler CB. Spontaneous rupture of the renal collecting system during pregnancy: successful management with a temporary ureteral catheter. J Urol. 1988;140(3):588-90.
10. Van Winter JT, Ogburn Jr PL, Engen DE, Webb MJ. Spontaneous renal rupture during pregnancy. Mayo Clinic Proceedings. 1991;66(2):179-82.
11. Hamoud K, Kaneti J, Smailowitz Z, Kroll D, Barki Y. Spontaneous perinephric urinoma in pregnancy. Int Urol Nephrol. 1994;26(6):643-6.
12. Royburt M, Peled Y, Kaplan B, Hod M, Friedman S, Ovadia J. Nontraumatic rupture of the Kidney in Pregnancy – case-report and review. Acta Obstetricia Et Gynecologica Scandinavica. 1994;73(8):663-5.
13. Hwang SS, Park YH, Lee CB, Jung YJ. Spontaneous rupture of hydronephrotic kidney during pregnancy: Value of serial sonography. Journal of Clinical Ultrasound. 2000;28(7):358-60.
14. Nabi G, Sundeep D, Dogra PN, Ambika. Spontaneous rupture of hydronephrotic solitary functioning kidney during pregnancy. Int Urol Nephrol. 2001;33(3):453-6.
15. Satoh S, Okuma A, Fujita Y, Tamaka M, Nakano H. Spontaneous rupture of the renal pelvis during pregnancy: A case report and review of the literature. American Journal of Perinatology. 2002;19(4):189-95.
16. Jang SJ, Kang DI. Huge Perirenal Urinomas in a Woman during the Postpartum Period. Korean J Urol. 2006;47(2):217-9.
17. Lang EK, Earhart V. Renal rupture in a preeclamptic patient with subsequent abscess. J Urol. 2006;175(2):731.
18. Lo KL, Ng CF, Wong WS. Spontaneous rupture of the left renal collecting system during pregnancy. Hong Kong Medical Journal. 2007;13(5):396-8.
19. Ushioda N, Matsuo K, Nagamatsu M, Kimura T, Shimoya K. Maternal urinoma during pregnancy. Journal of Obstetrics and Gynaecology Research. 2008;34(1):88-91.
20. Sidra LM, Keriakos R, Shayeb AG, Kumar N, Najia S. Rupture renal pelvicalyceal system during pregnancy. Journal of Obstetrics and Gynaecology. 2005;25(1):61-3.
21. Huang E, Sayegh R, Craigo S, Chelmow D. Rupture of the renal pelvis associated with intravenous fluid bolus. Journal of Maternal-Fetal and Neonatal Medicine. 2002;11(5):345-6.
22. Machado L, Kehinde EO, Leven HO. Hydronephrosis of pregnancy leading to urinoma formation. Journal of Obstetrics and Gynaecology. 1996;16(4):251-3.
23. Tang MB, Shen KS, Lee CW, Chen JY, Yeh CH, Chu CH. Rupture of the renal pelvis following hydronephrosis and hydroureter after a cesarean section. Taiwanese journal of obstetrics & gynecology. 2009;48(2):190-2.
24. Matsubara S, Morita T, Saito Y, Sato S, Suzuki M. Non-traumatic rupture of the left upper urinary tract during pregnancy without discernable underlying disorders. Arch Gynecol Obstet. 2010;282(1):111-3.
25. Efrimescu C BD, Mulvin D. Spontaneous Non-traumatic Upper Urinary Tract Rupture in Pregnancy: Case Report and Literature Review. Emergency Med. 2013;3(135).
26. Khandelwal A. Perinephric Urinoma in a Woman During the Postpartum Period: A Case Report. UroToday Int J. 2016;6(2).
27. Jalbani IK, Ather MH. Renal forniceal rupture in pregnancy secondary to obstructive renal stone presenting with acute renal failure. Saudi journal of kidney diseases and transplantation : an official publication of the Saudi Center for Organ Transplantation, Saudi Arabia. 2014;25(5):1081-3.
28. Kumar A SS, Khan I, Pridarshi S. Spontaneous Perinephric Urinoma in a Postpartum Woman: Case Report and Review. International Journal of Scientifi c Study. 2014;2(6):3.
29. Upputalla R, Moore RM, Jim B. Spontaneous forniceal rupture in pregnancy. Case reports in nephrology. 2015;2015:379061.
30. Boekhorst F, Bogers H, Martens J. Renal pelvis rupture during pregnancy: diagnosing a confusing source of despair. BMJ Case Rep. 2015;2015.
31. Hanson B, Tabbarah R. Preterm Delivery in the Setting of Left Calyceal Rupture. Case reports in obstetrics and gynecology. 2015;2015:906073.
32. Narasimhulu DM, Egbert NM, Matthew S. Intrapartum Spontaneous Ureteral Rupture. Obstetrics and Gynecology. 2015;126(3):610-2.
33. Patel S, Pates J. Rupture of the Maternal Renal Calyx Seconday to Fetal Malpresentation A Case Report. Journal of Reproductive Medicine. 2015;60(3-4):175-7.
34. Fluke LM, Hoagland BD, Bedzis SM, Johnston MG. Spontaneous Renal Calyceal Rupture: A Rare Cause of an Acute Abdomen in Pregnancy. Am Surg. 2016;82(8):196-7.
35. Deshmukh Y AS, Kohli S, Pati S. Spontaneous bilateral perinephric urinoma in a postpartum woman Indian Journal of Basic and Applied Medical Research. 2016;6(1):4.
36. Travieso J, Young OM. Antenatal Spontaneous Renal Forniceal Rupture Presenting as an Acute Abdomen. Case Rep Med. 2018;2018:8596491-.
37. Chen Y, Yan YF, Zhang Y, Carroll X, Li HR, Tao L, et al. Perinephric urinoma following spontaneous renal rupture in the third trimester of pregnancy: A case report and brief review of the literature. BMC Pregnancy and Childbirth. 2019;19(1).
38. Verhaeghe C, Panayotopoulos P, Descamps P, Legendre G. First case of spontaneous rupture of the left ureter in immediate post-partum. Journal of Gynecology Obstetrics and Human Reproduction. 2019;48(9):775-9.
39. Rikard-Bell A, Lockhart K, Malouf D, Karantanis E. What not to expect when you're expecting – Postpartum proximal ureteric rupture: A case report. Case Reports in Women's Health. 2020;26.
