# Supplementary material for: Spontaneous renal fornix rupture in pregnancy and the post partum period: a systematic review of outcomes and management
Source: BMC Urol. 2020 Aug 4;20:116. doi: 10.1186/s12894-020-00660-z (PMC7405429; doi:10.1186/s12894-020-00660-z)
Supplement: Supplementary file 2 — Additional file 2. [file 12894_2020_660_MOESM2_ESM.docx]

| **Pubmed** | **Embase** | **Cochrane library** | **Web of Science** | **Scopus** |
| --- | --- | --- | --- | --- |
| 341 results | 710 results | 176 results | 313 results | 39 results |
| #1 ((((((perineph*) OR (((((Kidney[MeSH Terms]) OR Ureter[MeSH Terms]) OR renal pelvis[MeSH Terms]) OR *caly*) OR *forni*)) OR urinary tract[MeSH Terms])) OR periren*) | 'kidney'/exp OR 'ureter'/exp  OR 'kidney pelvis'/exp OR  caly*:ti OR forni*:ti  OR ‘urinary tract’/exp or ‘renal’:ti | MeSH descriptor: [Kidney] explode all trees OR MeSH descriptor: [urinary tract] explode all trees | #1 TS=(ureter) OR TS=(renal pelvis) OR  TS=(kidney pelvis) OR TS=(*caly*) OR  TS=(*forni*) OR TS=(kidney) OR  TS=(perineph*) OR TS=(periren*) OR TS=(urinary tract) | #1 Kidney (key) OR Ureter (key)  OR “kidney pelvis” (ti,ab,key)  OR “renal pelvis” (ti,ab,key) |
| #2 (urinoma) OR extravas*) OR Ruptur* | 'urinoma' OR extravas*  OR ruptur* | MeSH descriptor: [ureter] explode all trees | #2 TS=(urinoma) OR TS=(extravas*) OR TS=(ruptur*) | #2 TITLE-ABS-KEY (*caly*  OR  periren*   OR  perineph*  OR  forni* ) |
| #3 (pregnan*) OR maternal | pregnan* OR maternal | MeSH descriptor: [Kidney Pelvis] explode all trees | #3 TS=(pregnan*) OR TS=(maternal) | #3 TITLE-ABS-KEY ( urinoma  OR   extravas*  OR  ruptur* ) |
| #1 AND #2 AND #3 | #1 AND #2 AND #3 | *caly* OR periren* OR perineph* | #1 AND #2 AND #3 | #4 TITLE-ABS-KEY ( pregnan*   OR  maternal ) |
|  |  | *Forni* |  | #5 #1 AND #2 AND #3 AND #4 |
|  |  | #1 or #2 or #3 or #4 or #5 |  |  |
|  |  | urinoma |  |  |
|  |  | extravas* |  |  |
|  |  | ruptur* |  |  |
|  |  | #7 or #8 or #9 |  |  |
|  |  | pregnan* |  |  |
|  |  | maternal |  |  |
|  |  | #11 or #12 |  |  |
|  |  | #6 and #10 and #13 |  |  |

Supplement 2: Search strategy used by authors for identification of possible studies for inclusion
